# Patients’ Perceptions of Pharmacogenetic Testing and Access to Their Results: State of the Art in Spain and Systematic Review

**DOI:** 10.3390/jpm12020270

**Published:** 2022-02-12

**Authors:** Pablo Zubiaur, David Nicolás Prósper-Cuesta, Jesús Novalbos, Gina Mejía-Abril, Marcos Navares-Gómez, Gonzalo Villapalos-García, Paula Soria-Chacartegui, Francisco Abad-Santos

**Affiliations:** 1 Clinical Pharmacology Department, Hospital Universitario de La Princesa, Instituto Teófilo Hernando, Universidad Autónoma de Madrid (UAM), Instituto de Investigación Sanitaria La Princesa (IP), 28006 Madrid, Spain; david97.prosper@gmail.com (D.N.P.-C.); jesus.novalbos.esterno@salud.madrid.org (J.N.); ginapaola.mejia@scren.es (G.M.-A.); marcos.navares@salud.madrid.org (M.N.-G.); g.villapalos@salud.madrid.org (G.V.-G.); paula.soria.externo@salud.madrid.org (P.S.-C.); 2 Centro de Investigación Biomédica en Red de Enfermedades Hepáticas y Digestivas (CIBERehd), Instituto de Salud Carlos III, 28006 Madrid, Spain

**Keywords:** pharmacogenetics, precision medicine, patients’ perceptions

## Abstract

The process of clinical pharmacogenetics implementation depends on patients’ and general population’s perceptions. To date, no study has been published addressing Spanish patients’ opinions on pharmacogenetic testing, the availability of the results, and the need for signing informed consent. In this work, we contacted 146 patients that had been previously genotyped at our laboratory and 46 healthy volunteers that had participated in a bioequivalence clinical trial at the Clinical Pharmacology Department of Hospital Universitario de La Princesa and consented to pharmacogenetic testing for research purposes. From the latter, 108 and 34, respectively, responded to the questionnaire (i.e., a response rate of 74%); Participants were scheduled for a face-to-face, telephone, or videoconference interview and were asked a total of 27 questions in Spanish. Great or almost complete acceptance of pharmacogenetic testing was observed (99.3%), age and university education level being the main predictors of acceptance rates and understanding (multivariate analysis, *p* = 0.004, R^2^ = 0.17, age being inversely proportional to acceptance rates and understanding and university level being related to higher acceptance rates and understanding compared to other education levels). Mixed perceptions were observed on the requirement of written informed consent (55.6% in favor); therefore, it seems recommendable to continue requesting it for the upcoming years until more perceptions are collected. The majority of participants (95.8%) preferred storing pharmacogenetic results in medical records rather than in electronic sources (55.6%) and highly agreed with the possibility of carrying their results on a portable card (91.5%). Patients agreed to broad genetic testing, including biomarkers unrelated to their disease (93.7%) or with little clinically relevant evidence (94.4%). Patients apparently rely on clinician’s or pharmacogeneticist’s interpretation and seem, therefore, open to the generation of ethically challenging information. Finally, although most patients (68.3%) agreed with universal population testing, some were reluctant, probably due to the related costs and sustainability of the Spanish Health System. This was especially evident in the group of patients who were older and with a likely higher proportion of pensioners.

## 1. Introduction

The process of clinical pharmacogenetics implementation at the national level in Spain is in full expansion. In 2019, the Spanish Senate concluded the need for the creation of a Clinical Genetics Hospital specialty to promote personalized precision medicine in the national health system [1]. Although, as of January 2022, it is not yet established, it is expected to happen soon, and there are numerous initiatives aimed at the implementation of pharmacogenetics in the country. In other countries, the situation is different, and there are institutions that issue applicable clinical pharmacogenetic guidelines. Of note are the Clinical Pharmacogenetics Implementation Consortium, United States, and the Dutch Pharmacogenetics Working Group, the Netherlands. These institutions are likely the pioneers in pharmacogenetics implementation activities worldwide. At the national level, in Spain, it is worth highlighting the Personalized Medicine Strategy of the Spanish Society of Pharmacogenetics and Pharmacogenomics (SEFF), which promotes the drafting and publication of pharmacogenetic clinical guidelines in Spanish to facilitate the implementation of the discipline in Spain and Latin American countries (information available at: https://seff.es/ (accessed on 11 December 2021)). These guidelines could be useful for around 500 million Spanish-speaking people or for the health professionals who treat them. In the Comunidad de Madrid, Spain, La Princesa University Hospital Multidisciplinary Initiative for the Implementation of Pharmacogenetics (PriME-PGx) [2] (promoted by the Clinical Pharmacology Department of Hospital Universitario de La Princesa) aims to implement pharmacogenetics at a regional level, in the region of Madrid, giving support to nearby areas. This initiative aims to promote pharmacogenetic routine testing in daily practice and to increase the availability of pharmacogenetic knowledge for any potential therapy.

Despite the importance of promoting these initiatives by pharmacogenetics tests providers, it is key to also understand “end-users’” (i.e., patients’) expectations. Ultimately, a pharmacogenetic test can be understood as purely diagnostic information and also as information that is proprietary to the patient. Such approaches were perhaps not pursued a few years ago because there were no strong stakeholders aimed at structuring the discipline in our country. However, nowadays, some ethical questions arise, and the patient’s perception is absolutely essential to our understanding. In this context, a paradoxical situation occurs: while there are several publications dealing with the opinions of healthcare personnel regarding pharmacogenetics [3,4], very few studies draw attention to the real protagonists of these tests, the patients. Hence, the primary objective of the present work was to address the Spanish general population’s opinion on pharmacogenetic testing, the availability of the results, and the need for signing informed consent.

## 2. Materials and Methods

### 2.1. Study Design, Procedures, and Population

The present work was a cross-sectional, observational study based on the completion of a questionnaire to two cohorts: one of patients who had received pharmacogenetic tests and the other of healthy volunteers enrolled in a bioequivalence clinical trial at the Clinical Pharmacology Department of Hospital Universitario de La Princesa who consented pharmacogenetic testing for research purposes. The reason we recruited the two cohorts is that, in our hospital, the patients are significantly older than the healthy volunteers in our clinical trial unit and have different sociodemographic characteristics. Therefore, we decided to include both cohorts so that the final sample would be more representative of the general population.

Inclusion criteria were as follows: adults aged between 18 and 75 years old who consented to participate in the study and complete the questionnaire; patients who had been previously requested a pharmacogenetic test by their practitioner as part of routine care; or healthy volunteers who had been enrolled in a bioequivalence clinical trial and provided informed consent for pharmacogenetic testing. Exclusion criteria included if, for reasons of age or cognitive ability, it was not possible to complete the questionnaire or if there was no response to the telephone after at least 4 calls spaced over the day.

Participants were scheduled for a face-to-face, telephone, or videoconference interview. A series of questions were asked, divided into the following sections: (A) informed consent and initial assessment of patient’s knowledge of pharmacogenetic testing; (B) demographic characteristics; (C) participant’s perceptions on pharmacogenetic testing, reporting, data storage, magnitude of testing consent; and (D) two open-ended questions. Supplementary Document 1 shows all questions translated into English of the whole questionnaire. Participants were provided with an explanatory text on pharmacogenetics, which is shown translated into English in Supplementary Document 2, required to answer the A2 and A3 questions (Supplementary Document 1).

### 2.2. Sample Size and Statistical Analysis

The GRANMO tool (available at: https://www.imim.es/ofertadeserveis/software-public/granmo/ (accessed on 11 December 2021)) was used to calculate the required sample size. Assuming a maximum disparity of opinion on the questions of 60% for “yes” and 40% for “no” (i.e., an expected proportion in the general population of 60%), a 95% confidence level, a replacement rate of 10%, and a precision of ±10%, a sample size of 103 subjects randomly selected was considered sufficient. In addition, because of the easy access to healthy volunteers participating in clinical trials at Unidad de Ensayos Clínicos del Hospital Universitario de La Princesa (UECHUP), we considered recruiting 50 additional healthy volunteer participants. As mentioned earlier, the aim for having two cohorts was to have a final cohort more representative of the general population in terms of age and sociodemographic characteristics.

A descriptive analysis of the responses to each question was performed, noting the percentage of patients who answered affirmatively. For the comparisons of responses between different groups (e.g., males vs. females, patients vs. healthy volunteers), a univariate analysis was performed with a Chi-squared test for qualitative variables, and Student’s *t*-test for quantitative variables was performed. Values of *p* < 0.05 were considered statistically significant. Afterward, a multivariate analysis was conducted by means of linear or logistic regression, where variables showing *p* < 0.05 in the univariate analysis along with demographic characteristics were included. All tests were conducted in SPSS v23 (IBM Corporation).

### 2.3. Ethics

Prior to completing the questionnaire, informed consent was requested verbally, and the approval was recorded in the questionnaire as the first question. No data were collected from patients who refused to participate. Identifying data were protected by assigning a number to each subject, which was recorded and stored in a file to which only the investigators of the study could access. Therefore, coded data were used, and confidentiality was maintained at all times. The research was conducted according to biomedical research and data protection European and Spanish laws. The project was approved by the Independent Ethics Committee of Hospital Universitario de la Princesa on 10 December 2020 (registration number 4322).

### 2.4. Systematic Review

Previous works documenting similar research activities (i.e., addressing patients’ perceptions on pharmacogenetic testing) were searched using PubMed, following a systematic methodology. The following search was conducted:

(“patient s” [All Fields] OR “patients” [MeSH Terms] OR “patients” [All Fields] OR “patient” [All Fields] OR “patients s” [All Fields]) AND (“percept” [All Fields] OR “perceptibility” [All Fields] OR “perceptible” [All Fields] OR “perception” [MeSH Terms] OR “perception” [All Fields] OR “perceptions” [All Fields] OR “perceptional” [All Fields] OR “perceptive” [All Fields] OR “perceptiveness” [All Fields] OR “percepts” [All Fields]) AND (“pharmacogenetics” [MeSH Terms] OR “pharmacogenetics” [All Fields] OR “pharmacogenomic” [All Fields] OR “pharmacogenomics” [All Fields] OR “pharmacogenomically” [All Fields])

A total of 167 articles were retrieved. Among them, most addressed physicians’ or pharmacy students’ perceptions of pharmacogenetic testing and were thus excluded from the initial screening; several others were not related to the topic; finally, 13 articles were included for evaluation. After review, 11 articles were outlined, as two of them were included in error and did not really present relevant studies.

## 3. Results

In total, 160 patients were screened, 146 fulfilled the inclusion criteria and were contacted, and 108 responded to the questionnaire, i.e., a response rate of 73.97%. As for the cohort of healthy volunteers, 46 subjects were contacted, and 34 responded, i.e., a response rate of 73.9%. Therefore, 142 participants were enrolled for the present study.

The initial assessment of patients’ knowledge of pharmacogenetic testing is shown in Table 1. Healthy volunteers reported a higher level of understanding of the test and greater confidence in explaining the procedure to a family member or friend (mean score on a scale of 1–10 of 8.89 vs. 8.25, respectively, *p* = 0.038 and 8.26 vs. 7.43, respectively, *p* = 0.009). Mean age in the patient group was significantly higher than in the group of healthy volunteers (*p* < 0.001). Most patients were born in Spain (77.8%); the prevalence of nationalities other than Spanish was significantly higher in the group of healthy volunteers (*p* < 0.001). All patients and no healthy volunteers suffered from conditions related to regular hospital visits (*p* < 0.001) (Table 2). Multivariate analysis revealed that age and the maximum study level of education were the main predictors of test understanding (*p* = 0.004, R^2^ = 0.17).

Regarding patients’ and healthy volunteers’ perceptions of pharmacogenetic testing, no significant differences were observed between the two groups on most questions. The vast majority of participants (99.3%) considered pharmacogenetic testing beneficial; mixed perceptions were observed on the requirement of signed consent for this type of testing (44.4% in favor). Furthermore, 97.2% of participants considered it appropriate to undergo additional pharmacogenetic biomarker testing beyond the specific one related to their current disease or treatment. In relation to the latter, mixed perceptions were observed on the informed consent request, with a similar result to the informed consent request for a general pharmacogenetic test (45.1% vs. 44.4%, respectively, *p* > 0.05). In addition, 98.6% of participants considered that they should be informed about results relevant to the management of their disease and 93.7% also about results relevant to another disease they might have in the future. No differences were observed between patients and healthy volunteers in any of the above questions (Table 2).

In terms of data storage and accessibility, the majority of participants agreed that these data should be stored or retained (97.9%) and included in their medical records (95.8%), with no differences between patients and healthy volunteers. Interestingly, significantly fewer patients agreed that their data should be stored on electronic servers, with them being the only ones with access to the data, compared to healthy volunteers (76.8% vs. 91.2%, *p* = 0.021). Multivariate analysis revealed that it was the age the true predictor for the response to this question (*p* < 0.0001, R^2^ = 0.124). Furthermore, the percentage of participants supporting the storage of these data on online servers that were accessible to healthcare staff was 55.6%; when asked about their perception of this on the basis of consent, the percentage increased to 91.2%. No differences were observed between patients and healthy volunteers for the latter perceptions. A slightly lower percentage of patients agreed to the storage of pharmacogenetic results on a portable card compared to healthy volunteers (88.9% vs. 100%, *p* = 0.066). Most patients agreed to undergo testing even if some results may not be useful today, assuming that some of them might be useful in the future (94.4%). Finally, 68.3% of the participants considered that this test should be done for any patient receiving medical care so that these results would be available in the future (Table 2).

Concerning open-end questions (D1, D2), 16 participants answered these questions (14 from the patient cohort and 2 from the healthy volunteer cohort). All responses focused on increasing awareness of the existence of these tests. Eight of them advocated raising awareness among healthcare professionals themselves through information campaigns in non-medical careers such as nursing, in services other than Clinical Pharmacology and primary health centers. Two commented that it would be useful to raise awareness among patients so that they themselves could spread the use of these tests. Four participants advocated that these tests should be explained to them in a clear and simple way. One of them suggested that this information should appear in electronic media such as the hospital’s website; one participant indicated these tests should be taught at the school in the biosanitary branches.

Table 2 shows the list of references to relevant works published to date addressing patients’ perceptions on pharmacogenetic testing. Most of them were promoted by institutions in the United States except for three, which included one or more of the following countries: Iceland, Denmark, United Kingdom, or Canada.

## 4. Discussion

Clinical pharmacogenetics implementation is a contemporary topic. In recent years, international efforts and resources have been devoted to the progress of the discipline. With this work, we aimed to describe the general population’s perceptions of pharmacogenetic testing in inhabitants of the area of Madrid, Spain; to the best of our knowledge, this is the first work that assesses such perceptions in our country and, in general, in Spanish-speaking countries.

In the present work, the fact that all participants achieved a sufficient degree of understanding of the pharmacogenetic test may be explained by the fact that it was a requirement to proceed to the next questions. If the participants did not understand it the first time, explanations were repeated, and eventually, they all understood it. Moreover, as they were patients whose management had previously depended on pharmacogenetic tests or healthy volunteers who had also been previously tested, the understanding of the text was probably faster than in a general population. Furthermore, great acceptance of pharmacogenetic testing was observed, which is consistent with earlier studies (Table 2). 

The differences in nationality and in each group are explained as follows: while the patients are mainly Spanish inhabitants of the surroundings of the Hospital Universitario de La Princesa, the volunteers can participate voluntarily and according to their economic needs, and the country of origin cannot be an inclusion or exclusion criteria. No differences were observed between patient and healthy volunteer groups, in contrast with earlier studies, where patients with mental disorders had more concerns on these tests than healthy volunteers or patients prescribed carbamazepine [12]. A previous study proposed that the degree of acceptance of such tests is directly proportional to the ability to accept and understand these studies [6]; moreover, patients with mental illness may be less willing to understand this type of testing because the management of their illness may have brought them stigmatization throughout their lives. One more test in which they can be classified as “good” or “bad” patients (from the point of view of therapy response) might seem to stigmatize them. Here, healthy volunteers had higher positive response rates to all but one (C5) question. Consistently, healthy volunteers showed a significantly higher level of understanding of the test than patients. Moreover, in several studies conducted in the United States, patients were more reluctant to undergo pharmacogenetic testing if this increased the cost of pharmacotherapy (Table 2). In this case, this was not assessed due to the free and universal nature of the Spanish National Health System. Moreover, a previous study indicated that only educational level is related to the degree of understanding of these tests [16]; here, age and the educational level were identified as the main predictors of understanding of these tests. This is consistent: older patients or those without a university education would be less likely to understand the intent of these tests (actually, all of them are capable of understanding them, but some better than others).

To our knowledge, this is the first work to assess patients’ perceptions of the requirement for informed consent for this kind of test. Surprisingly, only 44.4% of the participants considered it necessary to sign a written informed consent, which means that 56.6% would undergo these tests with oral or even without consent. This raises the question: can DNA be considered something owned by each person but managed by a clinician, just as organs are? For instance, a hepatologist may ask for a blood test to determine the liver function; can DNA genotyping be understood similarly? It seems that this is the perception of the population. However, as our genotyping technology yields additional results beyond the clinically useful ones, the ethics committee of our hospital considered it necessary to ask for the patient’s consent. Consistently, in accordance with the principle of Autonomy, it seems reasonable to ask for consent whenever there is the slightest doubt.

The high degree of support for information storage is consistent with the Spanish population’s solidarity with health issues, such as the very high rate of organ donors [21] and the public and universal nature of the system. As expected, there were significant differences in the degree of acceptance of storing this information on online servers. Logically, older, and therefore less technologically savvy, patients were more reluctant than younger, capable, healthy volunteers. Their main concern (data not shown) was the lack of safety of storing this type of data on online servers. The preferred database for storing these results was the electronic medical record, probably because it was the one they were most familiar with. However, it would be of great interest if pharmacogenetic data obtained in one hospital did not remain only there but could be exported to other centers where patients receive medical care. For instance, a patient with coronary disease, genotyped for *CYP2C19* (and all the other genes) prior to the surgery, could eventually be receiving medical care in their primary health center; in addition, this patient could be receiving simvastatin to control cholesterol blood levels; providing the physician at the primary health center with a set of clinically relevant pharmacogenetic information (in this case, SLCO1B1 phenotype would be the relevant biomarker) would signify better management of this patient. In this respect, we were positively surprised by the high degree of acceptance of the idea of storing these data on a portable card. In fact, every patient in Spain has a physical and electronic health card that could be a great repository for the results.

Surprisingly, there was a high level of acceptance for the genotyping of biomarkers other than the one required for their current medical condition, including those related to other diseases or biomarkers with no proven clinical relevance yet. This can probably be explained because patients feel that their clinician will interpret what is clinically relevant, and this is what they will be informed of. This, again, supports the theory that pharmacogenetic testing can be interpreted as a purely clinical test rather than the disclosure of something that is proprietary to the patient. May DNA genotyping, therefore, be compared to the measurement of another biomarker such as blood glucose? Or are there any additional ethical barriers? It seems this issue is of null importance for patients, probably because they think this will save them future collections or hospital visits in the future. 

The Spanish healthcare system is universal and publicly financed through taxation. Including pharmacogenetic testing in routine clinical practice would certainly increase the direct costs; therefore, the increase in public spending brings with it concerns from a large part of the population who do not want to pay more taxes. Sustainability is the balance between the two concepts: the health system should be the best possible with a reasonable amount of taxation and the lowest possible cost, which includes the system being efficient and not incurring unnecessary costs. In this context, the majority of participants agreed that this test should be universal for everyone (68.3%), but a significant proportion of them was reluctant, probably because of the costs involved and the sustainability of the health care system. Although it did not reach statistical significance, a higher percentage of patients were reluctant to the universal test compared to healthy volunteers. This is probably due to the fact that the sustainability of the system is of greater importance for the group of participants with the highest number of pensioners, which is the group of patients of greater age.

However, the implementation of clinical pharmacogenetics depends on several factors in addition to patients’ perception of routine testing. To our knowledge, these factors can be summarized as (a) lack of consistency in clinical recommendations, (b) budgetary constraints, (c) lack of well-trained pharmacogeneticists, and lack of commitment of healthcare personnel (mainly physicians and pharmacists) [2,22]. Regarding the first limitation, we seem to be moving in the right direction, with institutions such as the CPIC and DPWG issuing pharmacogenetic guidelines in English (and Dutch) and with the SEFF issuing pharmacogenetic guidelines in Spanish. Regarding the second limitation, studies demonstrating the cost-effectiveness of pharmacogenetic testing are warranted; however, the solution to this limitation may vary significantly by country. In countries where healthcare costs are mainly privately financed, companies offering commercial tests are involved in the development of pharmacogenetic tests [22]. By contrast, in countries with public health systems, these companies are less frequent because these tests are usually performed by the Clinical Analysis, Pharmacology, or Pharmacy departments of public hospitals (or at least that is the model towards which progress is being made). Therefore, the solution to the second limitation depends, for the first type of country, on insurance companies offering it in competitive and affordable packages for the patient, while for the second type of country, the second point depends on the sustainability of the system. As for the third limitation, efforts aimed at training pharmacogeneticists are warranted. By forming part of multidisciplinary teams in hospitals, this figure would allow the training of the healthcare personnel involved (mainly physicians and pharmacists). In addition, specific periodic training actions are recommended. Our initiative is currently promoting a project similar to the present one, in which the level of training in pharmacogenetics of hospital personnel will be collected and free periodic training will be offered.

## 5. Conclusions

Clinical pharmacogenetics implementation must be guided by patients’ perceptions and not only by clinicians’ or pharmacogeneticists’ preferences. To our knowledge, the present work is the first one of its kind in the Spanish population published to date. Almost complete acceptance of pharmacogenetic testing was observed, age being inversely proportional to acceptance rates and understanding and university level being related to higher acceptance rates and understanding compared to other education levels. Mixed perceptions were observed on the requirement of written informed consent; therefore, it seems recommendable to continue requesting it for the upcoming years until more perceptions are collected. The majority of participants preferred storing pharmacogenetic results in medical records rather than in electronic sources and highly agreed on the possibility of carrying their results on a portable card. Patients agreed to broad genetic testing, including biomarkers unrelated to their disease or with little clinically relevant evidence. Patients apparently rely on clinician’s or pharmacogeneticist’s interpretation and seem, therefore, open to the generation of ethically challenging information. Finally, although most patients agreed with universal population testing, some were reluctant, probably due to the related costs and sustainability of the Spanish Health System. This was especially evident in the group of patients who were older and with a likely higher proportion of pensioners. As a final conclusion, the degree of understanding and acceptance of pharmacogenetic testing is essential for the implementation of precision medicine, both in our hospital and in any other in the world. Therefore, initiatives aimed at training patients in hospitals are necessary in addition to other parallel initiatives, such as the training of healthcare personnel or staff in training.

## 6. Highlights

- The Spanish population understands and highly accepts pharmacogenetic testing, with the level of understanding being proportional to educational level and inversely proportional to age. Unlike other countries with private or mixed healthcare systems, the cost of pharmacogenetic testing does not generally seem to be a concern for them because it is assumed by the system and not by them; however, the concern for universal population testing increases with age.
- The Spanish population has great confidence in health personnel and has no objection to generating genetic information beyond what is necessary at the present time.
- The electronic medical record at the hospital is the preferred means of storing genetic data for the Spanish population, who would also welcome it if these data could be uploaded onto their health card or a similar means.
- Patients apparently do not feel it is necessary to sign a written informed consent but would rather consent to their physician as part of routine clinical practice, as if they had a blood test. However, for broad genetic studies, requesting the signature of informed consent seems prudent until more insights are collected in the future.

## Figures and Tables

**Table 1:** Results of the questionnaire.

Question ID	Question	Total (*n* or Mean (% or SD))	Patients (*n* or Mean (% or SD))	Healthy Volunteers (*n* or Mean (% or SD))	*p*
(A) INITIAL ASSESSMENT					
A1	Do you consent to being asked the following questions?	146 (100%)	108 (100%)	34 (100%)	1.000
A2	Do you know what a pharmacogenetic test is and what it is for? If your answer is “no”, we will explain what it is and let you ask questions.				
A3	Have you already understood what a pharmacogenetic test is and what it is used for? If not, we will explain it to you again until you understand the test.				
A4	On a scale from 1 to 10, with 10 being absolute understanding, how well do you think you understood?	8.40 (1.29)	8.25 (1.36)	8.89 (0.86)	0.038
A5	On a scale from 1 to 10, with 10 being absolute confidence, how confident would you be in explaining this procedure to your family or friends?	7.63 (1.82)	7.43 (1.93)	8.26 (1.23)	0.009
(B) DEMOGRAPHIC CHARACTERISTICS					
B1	Age	50.9 (18.6)	56.5 (17.1)	33.2 (9.9)	<0.001
B2	Male sex	66 (46.5%)	47 (43.5%)	15 (44.1%)	0.951
B3	Born in Spain	92 (64.8%)	84 (77.8%)	8 (23.5%)	<0.001
B4	Level of education: university	77 (54.2%)	56 (51.9%)	21 (61.8%)	0.312
B5	Any kind of professional relationship with healthcare sector	24 (16.9%)	22 (20.3%)	2 (5.9%)	0.067
B6	Illness that requires regular hospital visits	108 (76.1%)	108 (100%)	0 (0%)	<0.001
(C) PARTICIPANT’S PERCEPTIONS					
C1	Do you consider these tests to be useful or beneficial?	141 (99.3%)	107 (99.1%)	34 (100%)	1.000
C2	Do you think patients can undergo these tests without written consent?	63 (44.4%)	47 (43.5%)	16 (47.1%)	0.717
C3	Do you think that if you require testing for a particular gene, you should also be tested for other genes that you may or may not require in the future?	138 (97.2%)	104 (96.3%)	34 (100%)	0.572
C4	Do you consider it necessary to sign an informed consent form in the case of the previous question?	64 (45.1%)	48 (44.4%)	16 (47.1%)	0.891
C5	Do you think you should be informed of results that are useful for the management of your current disease?	140 (98.6%)	107 (99.1%)	33 (97.1%)	0.423
C6	Do you think you should also be informed of results that are not useful for the treatment of your current disease but could be useful for other diseases you may have in the future?	133 (93.7%)	100 (92.6%)	33 (97.1%)	0.687
C7	Do you agree to the retention or storage of the data obtained?	139 (97.9%)	105 (97.2%)	34 (100%)	1.000
C8	Do you agree to have these data included in your medical records in a similar way as if you have an allergy to any medication?	136 (95.8%)	102 (94.4%)	34 (100%)	0.336
C9	Do you agree if these data are stored on an electronic server to which only you have access?	109 (76.8%)	78 (72.2%)	31 (91.2%)	0.021
C10	Do you agree to have these data stored on an electronic server that can be accessed by health personnel?	79 (55.6%)	57 (52.8%)	22 (64.7%)	0.222
C11	Do you agree to have these data stored on an electronic server that can be accessed by health personnel but only with your consent?	130 (91.5%)	97 (89.8%)	33 (97.1%)	0.294
C12	Would you like these data to be stored on a card that you could carry with you and show to health personnel when required?	130 (91.5%)	96 (88.9%)	34 (100%)	0.066
C13	Do you agree to be tested knowing there are results that are not useful today but may be useful in the future?	134 (94.4%)	100 (92.6%)	34 (100%)	0.198
C14	Do you think this test should be done to everyone, so the results are available in the future if needed?	97 (68.3%)	71 (65.7%)	26 (76.5%)	0.241

Data are presented as the number and % of affirmative responses for questions with yes/no answers; for questions whose response is a continuous variable, the mean and standard deviation are provided; *p* values correspond to the univariate analysis.

**Table 2:** Relevant works published to date addressing patients’ perceptions on pharmacogenetic testing.

Reference	Country	Sample	Main Conclusion
Almarsdottir et al., 2005 [5]	Iceland, Denmark	42 adults	Participants were concerned about drugs developed based on pharmacogenomics being more expensive than conventional mass-produced drugs, which would lead to inequalities locally and globally
Sanderson et al., 2008 [6]	United Kingdom	1024 adults	Most participants were interested in genetic testing; those who anticipated regret about genetic testing expressed lower interest than those who did not anticipate regret
Grant et al., 2009 [7]	United States	152 primary care patients and 89 diabetic patients enrolled in a pharmacogenetics study	Patients generally favored genetic testing for diabetes risk prediction; a “high risk” result would very likely improve motivation to adopt preventive lifestyle changes and treatment adherence
Madadi et al., 2010 [8]	United States	62 codeine-prescribed breastfeeding mothers participating in a study where CYP2D6 was genotyped	All participants wanted to receive the pharmacogenetic test result; they differed in the value of the usefulness of this information toward future medical decisions; 33% of the participants wanted to withhold these results from their physicians
De Marco et al., 2010 [9]	United States	34 African American and 14 White patients	In general, patients considered precision medicine and genetic testing to be positive advances in medicine, despite not having a clear understanding of what these practices entail; the White group expressed concern that the practice would be too expensive; the African American group was concerned that medical mistrust of marginalized populations might affect the acceptance of genetic testing
Zhang et al., 2014 [10]	Canada and United States	226 adult parents, 105 adult nonparents	Adequate explanation prior to pharmacogenetic testing was the most important issue for participants; those with greater knowledge of pharmacogenetics were also more comfortable with pharmacogenetic testing; when this test was for their children, parents valued their understanding more than their children’s opinion; most participants considered informed consent necessary for this type of testing
Lachance et al., 2015 [11]	Canada	175 healthy volunteers, 175 patients with heart failure, and 100 heart transplant recipients	Most participants stated that they would accept pharmacogenomic testing and expressed high hopes regarding its potential applications; healthy individuals were more concerned about potential employment and insurance discrimination and were more worried about confidentiality issues
Trinidad et al., 2015 [12]	United States	27 patients prescribed antidepressants, 17 patients prescribed carbamazepine, and 17 healthy patients	Most participants understood the potential advantages of pharmacogenetic testing; many of them felt that the risks (discrimination, stigmatization, physician overreliance on genomic results, and denial of certain medications) might outweigh the benefits. These concerns were more strongly expressed among participants with chronic mental health diagnoses
Lee et al., 2017 [13]	United States	9 pharmacogenomic and 13 traditional care patients	Participants experiencing pharmacogenomic-guided care were more receptive toward pharmacogenomic information being used than traditional care participants
McKillip et al., 2017 [14]	United States	507 patients	The perception of personalized care was significantly higher in patients with genomic guidance compared to patients without genomic guidance
Gibson et al., 2017 [15]	United States	27 patients	Patients were generally interested in pharmacogenetic testing, but with varying levels of willingness to pay; they would more likely use the service if their insurance covered the cost
Olson et al., 2017 [16]	United States	869 patients	Patients’ understanding of pharmacogenetic test results was low, with education level being the only predictor of understanding; most patients agreed that adherence to treatment would improve if pharmacogenetic information was used to guide prescription
Bright et al., 2020 [17]	United States	19 patients: 10 at rural location and 9 at urban location	Qualitative assessment of patients’ perceptions; trust, experience, risk-benefit, and clarity were the main themes that conditioned willingness
Asiedu et al., 2020 [18]	United States	24 patients	Different educational materials for training patients in pharmacogenetics were evaluated: a letter, a video, and a brochure. None of them were superior overall; however, patients were concerned about the amount of detail included in some materials and the use of overly technical language
Stancil et al., 2021 [19]	United States	17 adolescent patients	Adolescents understood the reason for pharmacogenetic testing, and most felt the results impacted their current andfuture care. None perceived risks to securing future employment or insurance. All felt pharmacogenetics would be beneficial in general
Saulsberry et al., 2021 [20]	United States	463 patients preventively genotyped for guiding pharmacotherapy	Self-reported Black patients were less confident about pharmacogenetic-guided decisions and wanted a principal role for their genetic information in clinical care. Self-reported White patients were more likely to discuss the impact of genetic results on medication response than Black patients

## Data Availability

All data are presented in this manuscript.

## Supplementary Materials

The following are available online at https://www.mdpi.com/article/10.3390/jpm12020270/s1, Supplementary Document S1. Questionnaire asked to study participants translated into English., Supplementary Document S2. Explanatory text.
