# Supplementary material for: Patients’ Perceptions of Pharmacogenetic Testing and Access to Their Results: State of the Art in Spain and Systematic Review
Source: J Pers Med. 2022 Feb 12;12(2):270. doi: 10.3390/jpm12020270 (PMC8879541; doi:10.3390/jpm12020270)
Supplement: Supplementary file 1 [file jpm-12-00270-s001.zip › Supplementary Document S1.pdf]

Supplementary Document S1. Questionnaire asked to study participants translated into English.

A) INITIAL ASSESSMENT	
**Question ID**	**Question**
**A1**	Do you consent to be asked the following questions?
**IF YOU DISAGREE, DO NOT REPLY ANY FURTHER**	
**A2**	Do you know what a pharmacogenetic test is and what it is used for? If you answer "no", we will explain what it is and let you ask questions
**A3**	Have you already understood what a pharmacogenetic test is and what it is for? If not, it is explained again until we are sure you understand the test.
**A4**	On a scale of 1 to 10, 10 being an absolute understanding of the test, how well do you think you understood it?
**A5**	On a scale of 1 to 10, 10 being absolute confidence, how confident are you in explaining this procedure to family or friends?
**B) DEMOGRAPHIC CHARACTERISTICS**	
**Question ID**	**Question**
**B1**	Age
**B2**	Sex
**B3**	Country of origin
**B4**	What was your highest level of education attained: no education, primary, secondary or university?
**B5**	Do you have an employment relationship with the health sector?
**B6**	If you have any illnesses that require regular hospital visits, can you tell us which ones?
**C) PARTICIPANT'S PERCEPTIONS**	
**Question ID**	**Question**
**C1**	Do you consider these tests to be useful or beneficial?
**C2**	Do you think patients can undergo these tests without written consent?
**C3**	Do you think that if you require testing for a particular gene, you should also be tested for other genes that you may or may not require in the future?
**C4**	Do you consider it necessary to sign an informed consent form in the case of the previous question?
**C5**	Do you think you should be informed of results that are useful for the management of your current disease?

**C6**	Do you think you should also be informed of results that are not useful for the treatment of your current disease but could be useful for other diseases you may have in the future?
**C7**	Do you agree to the retention or storage of the data obtained?
**C8**	Do you agree to have these data included in your medical records, in a similar way as if you have an allergy to any medication?
**C9**	Do you agree if these data is stored on an electronic server to which only you have access?
**C10**	Do you agree to have these data stored on an electronic server that can be accessed by health personnel?
**C11**	Do you agree to have these data stored on an electronic server that can be accessed by health personnel but only with your consent?
**C12**	Would you like these data to be stored on a card that you could carry with you and show to health personnel when required?
**C13**	Do you agree to be tested knowing there are results that are not useful today but may be useful in the future?
**C14**	Do you think this test should be done to everyone so the results are available in the future if needed?
**D) OPEN-ENDED QUESTIONS**	
**Question ID**	**Question**
**D1**	If you answered NO to any question and you want to let us know why, please comment in this section.
**D2**	What do you recommend or what would you do to facilitate the implementation of these tests and their acceptance in the population?
