# Supplementary material for: Patients’ Perceptions of Pharmacogenetic Testing and Access to Their Results: State of the Art in Spain and Systematic Review
Source: J Pers Med. 2022 Feb 12;12(2):270. doi: 10.3390/jpm12020270 (PMC8879541; doi:10.3390/jpm12020270)
Supplement: Supplementary file 1 [file jpm-12-00270-s001.zip › Supplementary Document S2.pdf]

Supplementary Document S2. **Explanatory text.**

The European Medicines Agency (EMA) defines pharmacogenetics as "the study of DNA sequence variations related to drug response", i.e., how small changes in DNA are responsible for the different results some drugs produce in different people. These small changes are known as polymorphisms and we can describe them using a blood sample and a technique known as array-based genotyping. These polymorphisms may cause a drug to be more likely to cause harm (e.g., an allergic reaction) or be less effective in certain patients, among other things. Pharmacogenetics aims to describe these associations in order to prescribe treatments that *a priori* should not cause problems.

For example, a mutation in a gene known as *HLA-B**5701 has been linked to a severe form of allergy, called a hypersensitivity reaction, in people taking abacavir (a drug used for the treatment of the HIV infection). This hypersensitivity reaction can range from mild symptoms such as diarrhea or skin spots to a life-threatening reaction. With this technique, we identify the carriers of the mutation, for which another drug is prescribed, avoiding the possibility of causing unnecessary harm. This is not its only advantage: it also reduces the cost to the healthcare system (and therefore to the state) of giving the incorrect treatment and the cost of treating the adverse drug reaction, not to mention the fact that we can prevent misfortunes such as death or hospitalization.

This happens with many other drugs, but like everything else in life, nothing is infallible. There are still adverse drug reactions that we cannot predict. There are polymorphisms that we know about but that, to date, we still do not know if they are useful and if we should include them in the clinical practice. Moreover, these tests are not performed on everyone and, in addition, the variations in drug response do not depend only on these changes in the DNA, but on many other things.
